# Towards opioid access without excess

**DOI:** 10.1016/S2468-2667(25)00035-0

**Published:** 2025-02-26

**Authors:** William E Rosa, Felicia Marie Knaul, Michael Touchton, Afsan Bhadelia, Keith Humphreys, Lukas Radbruch, M R Rajagopal

**Affiliations:** Department of Psychiatry and Behavioral Sciences, Memorial Sloan Kettering Cancer Center, New York, NY, USA; Department of Medicine, David Geffen School of Medicine, University of California, Los Angeles, Los Angeles, CA, USA; Institute for Advanced Study of the Americas, University of Miami, Coral Gables, FL, USA; Escuela de Medicina y Ciencias de la Salud, Technológico de Monterray Faculty of Excellence, Mexico City, Mexico; Tómatelo a Pecho, Asociación Civil, Mexico City, Mexico; Institute for Advanced Study of the Americas, University of Miami, Coral Gables, FL, USA; Department of Political Science, College of Arts and Sciences, University of Miami, Miami, FL, USA; Institute for Advanced Study of the Americas, University of Miami, Coral Gables, FL, USA; Department of Public Health, College of Health and Human Sciences, Purdue University, West Lafayette, IN, USA; Department of Psychiatry and Behavioral Sciences, Stanford University, Palo Alto, CA, USA; Center for Innovation to Implementation, Veterans Affairs Health Care System, Palo Alto, CA, USA; Department of Palliative Medicine, University of Bonn, Bonn, Germany; International Association for Hospice & Palliative Care, Houston, TX, USA; Trivandrum Institute of Palliative Sciences, Thiruvanthapuram, Kerala, India; Department of Palliative Care, Pallium India, Thiruvanthapuram, Kerala, India

## Abstract

A decade after World Health Assembly Resolution 67.19 recognised palliative care as a component of comprehensive care and universal health coverage throughout the life course, a balanced approach to opioid access remains elusive. Disparities in the alleviation of serious health-related suffering persist, characterised by two parallel opioid-related public health crises: a pandemic of unrelieved pain and an opioid addiction epidemic. The pain pandemic is largely driven by the opioid access abyss and primarily affects over 50 million people with serious health-related suffering living in low-income and middle-income countries. Conversely, several high-income countries have been affected by an opioid addiction epidemic. An estimated 39·5 million people struggle with opioid use disorder worldwide, the vast majority living in North America. In this Viewpoint, we describe these parallel crises, argue for pain relief as a global public good, and identify stewardship roles of WHO member states and local actors to use a balanced approach in galvanising the global health system to simultaneously close the global pain divide and move towards opioid access without excess in alignment with Sustainable Development Goal targets focused on the prevention of substance abuse (3.5) and universal health coverage (3.8).

## Introduction

World Health Assembly Resolution 67.19 established palliative care as a core component of comprehensive health care throughout the life course and of universal health coverage.^1^ This was the first formal recognition by WHO member states that palliative care provision is an ethical and financial responsibility of health-care systems. The Resolution called for multisector investments in health-care system strengthening with ongoing monitoring and evaluation of associated outcomes (eg, access to opioids). Importantly, World Health Assembly Resolution 67.19 prioritised the relief of physical pain, acknowledging that opioid access for clinical and research purposes remains inadequate in several countries and the prevention of illicit narcotic trafficking and diversion should not create insurmountable regulatory barriers to prescribed opioids.^1^

Actors such as the International Narcotics Control Board (INCB) have long promoted a balanced approach to availability of controlled essential medicines,^2-4^ calling for equitable access to medically and scientifically indicated opioids with policies and practices to counter opioid misuse and diversion.^5^ Balanced policy making recognises that excessive opioid use in some high-income countries (HICs) does not justify denying clinically appropriate access elsewhere, including in low-income and middle-income countries (LMICs).^6,7^

WHO has committed to a balanced approach through global solidarity with both state and non-governmental actors. In keeping with the World Health Assembly Resolution 67.19, WHO supports and advocates for enhancing supply chain coordination, strengthening governance, bolstering of financial and human resources, and addressing pervasive misconceptions about pain, addiction, and opioids. WHO upholds morphine as the low-cost, essential medicine for relieving moderate to severe pain—especially in LMICs—in alignment with the *Lancet* Commission on global access to palliative care and pain relief.^8^ Data consistently show that little headway has been made to address unrelieved pain since World Health Assembly Resolution 67.19,^8-11^ which means that calls for a balanced approach go unheeded.

Even COVID-19 failed to improve equitable pain relief.^12^ During the pandemic, WHO, INCB, and the United Nations Office on Drugs and Crime encouraged countries to expand their stocks of opioids and provided rapid-response facilities to support increased availability but success was limited and sustainability is unclear.^13^ WHO retracted two key international pain management guidelines in 2019, leaving health-care workers without evidence-based best practice documents throughout most of the pandemic.^14,15^ Calls for improved access in other types of emergencies (eg, armed conflict) have similarly fallen short.^16-19^

Given the inability to date to achieve a balanced approach, we aim to provide strategies to close the global pain divide without neglecting the equally dire opioid addiction epidemic.^20-23^

## Parallel crises: the pain pandemic and opioid addiction epidemic

Opioids have a dual nature: both oversupply and undersupply dramatically increase human suffering. The pain pandemic is epitomised by human-made scarcity and the opioid addiction epidemic is the epitome of oversupply. For the relief of pain and refractory breathlessness in many severe illnesses and at end of life, opioids are essential and, without them, palliative care clinicians, patients, and their loved ones lose a crucial tool.^24,25^ More than 70 million people worldwide with serious illness or injury experience health-related suffering that is amenable to palliative care, making the full range of universal health coverage elusive for almost all countries.^8,26,27^ The opioid access abyss affects about 80% of those in need—and primarily over 50 million people with serious health-related suffering living in LMICs.^8^ This access desert is primarily driven by poor policy making at national and global levels. The richest 10% of countries possess 90% of distributed opioid medicines, while the other 50% have access to only 1% of the opioids distributed annually.^8^ Pain layers onto poverty, as lack of opioid access primarily affects impoverished communities, and unrelieved suffering leads to disability; this in turn can generate a loss of income and financial catastrophe for patients and caregivers.

In stark contrast, an opioid addiction epidemic of oversupply has befallen several HICs, such as the USA, Canada, and Australia. An estimated 39·5 million people struggle with opioid use disorder globally.^28^ In the USA, this overdose epidemic started in the 1990s with rapidly expanded opioid prescriptions for a broad range of chronic non-cancer pain conditions, followed by a second wave largely propelled by increased usage of illicit heroin and fentanyl.^29^ A key lesson learned is that opioids are not a panacea for all pain conditions, and for some chronic pain syndromes (eg, headache or low-back pain) opioids should only be used as part of multimodal treatment regimens, or not at all.^30,31^

Valid concerns about the opioid addiction epidemic have stymied efforts to facilitate access to opioid medicines essential for palliative care across countries. For example, strict limits on opioid prescribing in some countries increase patient suffering from common medical afflictions and at end of life.^32,33^ Thus, the lack of a balanced approach is abandoning people in need amid two contemporary and interlocking global health crises—both an inadequate access to and excess of opioids.

## Closing the pain divide

The *Lancet* Commission on global access to palliative care and pain relief emphasised that inequitable access to opioids for the relief of health-related suffering in serious illness and at end of life is a moral travesty of global proportions.^8^ The market is organised around higher priced, on-patent opioid medicines, incentivising their purchase while the pharmaceutical industry limits morphine manufacturing because of low profit margins and high risk of liability.^34^ Hence, morphine is the least expensive opioid available to patients with low income and LMICs that at the same time has negligible street value.

The Stanford–*Lancet* Commission on the North American opioid crisis drew on these conclusions, noting that the profit motive of legal analgesic manufacturers should also be considered one of the access challenges in LMICs.^29^ This guidance was coupled with recommendations to fund evidence-based treatment plans for opioid addiction, advocate policies to treat addiction as a public health rather than criminal issue, provide safe opioid disposal pathways, and implement regulatory barriers in HICs to prevent pharmaceutical companies from pressuring LMICs to overprescribe on-patent opioids.^29^

The aforementioned *Lancet* Commissions^8,29^ focused their recommendations for pain relief on access to morphine as the off-patent, low-cost, most effective medicine for treating moderate to severe pain. Without financial incentives to produce and market generic morphine, policies are required to increase its supply and remove the pharmaceutical profit motive from the global pain relief ecosystem.^8,29^ And both Commissions focused on public health as the primary goal and agreed that eliminating the profit motive would substantially reduce overprescribing, key to achieving Sustainable Development Goal (SDG) 3.5 (prevention and treatment of substance abuse).^35^ The congruence of these two Commissions—comprised of experts analysing very different challenges—speaks to opportunities for achieving a balanced approach to opioid access and to the destructiveness of using oversupply as a rationale for undersupply.

Despite the justification for cheap, immediate-release oral morphine, a survey of opioid pricing found several countries with complex delivery formulations (eg, slow-release morphine, oxycodone, and transdermal fentanyl) included in their national essential medicine list, and often available at a lower price compared with immediate-release morphine.^34^ This observation raises concerns about the implications of subsidising opioids in some LMICs while not in others, thus generating price differentials between LMICs; and it also raises apprehensions about effective, equitable implementation of the global public good framework if intellectual property rights and rights of capital, and related decision making, cannot be questioned when it comes to ensuring public health access and protections for all countries.^36^

## Safe access to pain relief as a global public good

There is an urgent need to re-evaluate health-care policies and resource allocation surrounding opioid use globally. We propose that treating safe and equitable access to pain relief as a global public good is complimentary to the fundamental human rights rationale for alleviating human suffering and to the tenets of global health and the SDGs.^20,21,23,37^ In contrast to private goods, no one can be excluded from benefiting from a public good (ie, pain relief) and one person’s consumption does not prevent anyone else from using it.^20^

Because access to pain relief as a global public good addresses both the pain pandemic and opioid addiction epidemic, a balanced approach can only happen through global stewardship to catalyse the global health-care system and ameliorate both.^7,38^ For example, translating the Commission on global access to palliative care and pain relief^8^ findings to policy action is only possible through increased stewardship by international entities, bringing together WHO member states to not only ratify but also implement recommendations. Treating pain relief as a public health priority means guaranteeing that opioid access does not substantially increase illicit use, which is a negative externality of these policies. Such a pledge requires national health-care systems to act in tandem with the global health system. Although national policies are key to achieving balance between ensuring access and preventing excess of opioids, existing global controlled substance regulations—and the international production of morphine and other opioids coupled with the cross-border flow of medicines and illicit substances—require global collective action and stewardship guided by international regulations.^8,39^ Producers have international reach, leading to positive and negative effects, which can serve to expand access through increased opioid production but also serve to exert pressure on national actors and clinicians to overprescribe, respectively. Overprescription can be averted through a balanced approach.

Stewardship of global efforts for access to pain relief as a global public good must include building the evidence base through research, providing technical assistance, establishing normative functions, such as regulations and clinical guidance, and managing externalities.^40^ The externalities or spillover effects of expanded opioid access can be negative (eg, stigma around opioid use and potential inadvertent profit incentivisation) or positive (eg, strengthened regulatory frameworks, supply chain coordination, health workforce training, and medical decision making, as well as improved purchasing platforms, pooled procurement of medicines, and partnership between palliative care and substance addiction communities). The stewardship to address negative externalities and maximise positive externalities calls for close cooperation across international agencies, especially WHO and INCB, coordination with national and regional entities, and partnerships with the scientific community, civil society, and producers (private sector and other) to implement multilevel approaches that seek to address the dual challenges of lack of access to opioids for some and excess for others.

Too few international organisations have taken responsibility for promoting a balanced approach and prioritising key global public goods. WHO has long recognised morphine as the gold standard for pain relief, including it in the WHO Model List of Essential Medicines since 1977.^41^ This list is itself a global public good, complemented by expert-informed, evidence-based guidelines that are globally recognised as best practice documents.^25,42^ The INCB stands out for encouraging countries to expand their official, estimated requirements for opioid medicine in line with health needs (eg, as defined by serious health-related suffering).^2,41^ Other entities could provide key financing and technical assistance platforms, such as the World Bank and the regional development banks, which could play formidable roles in advancing access to pain relief. Using WHO-driven evidence as a lever,^10^ member states are now empowered to help close the global pain divide by promoting access without excess.

The inequities and inefficiencies in the pricing of morphine products results in LMICs facing higher prices than HICs.^8^ This inequity is at least partly related to the smaller market size and lower negotiating capacity of most LMICs. Global stewardship is key to organising purchasing platforms that aggregate demand and boost negotiating power across low-income and small countries. Successful purchasing platforms are in operation for other medicines and offer tiered pricing, such as through the Global Fund to fight AIDS, Tuberculousis and Malaria^43^ and the Pan American Health Organization’s Strategic Fund,^44^ which includes the opportunity to purchase morphine at favourable prices. Yet, these opportunities are too seldom availed of by countries.^45^ Moreover, subscription financing,^46^ which can offer patients access to medicines at a fixed price within a defined period of time, has shown promise, but, especially in LMICs, research on its use to improve opioid access is necessary.

The Commission on global access to palliative care and pain relief advanced a research agenda to guide universal access to palliative care and pain control and provide data and indicators for monitoring and evaluation.^8^ Generating and implementing better metrics (including refined metrics of both serious health-related suffering^26^ and distributed opioids in morphine equivalent as a global measure of palliative care availability)^47^ requires financial resources, sustained global stewardship (that would probably best be formed from WHO), and collaborative spaces for all actors. Innovative metrics to measure the burden of suffering—from both inadequate opioid access and opioid excess—and the value of alleviating suffering are key priorities. Existing mortality and morbidity metrics largely ignore suffering in patient and caregiver wellbeing. Thus, community-based research and policy endeavours must centre and amplify the lived experiences of communities.

Strengthening the evidence base for LMICs on the pain pandemic is of the highest priority. Almost 95% of the available medical evidence stems from HICs which account for only 15% of the global population.^48^ Reliance on existing evidence risks global guidelines being based on HIC needs while delaying restorative justice in LMICs.^49,50^ In addition, the relevant evidence is skewed towards opioid excess rather than access; about 90% of the literature related to opioid medicines focuses on opioid misuse and addiction whereas only 10% focuses on the dearth of opioid access globally.^51^ This imbalance does not imply that less research on opioid excess is needed, but rather, more empirical work on opioid access is a must.

Beyond the need for improved research, medical training, and clinical practices, member states must not only persist but go much deeper in providing clarity to WHO on national level needs and should recommend regulatory changes in alignment with a balanced approach. To date, very few countries prioritise the pain pandemic, and fewer formally advocate to the international community for relevant policy change. A balanced approach requires a balanced conversation between local, national, regional, and international actors. In the panel, we summarise our recommendations for WHO member states, international organisations, and local and global actors to realise opioid access without excess.

## Conclusion

Using the North American opioid addiction epidemic to establish policy decisions for a global population alleviates neither the pain pandemic nor the opioid addiction epidemic.^15^ We must galvanise the global health system to achieve and sustain opioid access without excess as a global public good. Strategies that unite actors around this goal (panel) can empower and enfranchise the most vulnerable, low-income, and sickest populations, and strengthen national and global health-care systems to achieve both SDG 3.5 (prevention of substance abuse) and SDG 3.8 (universal health coverage). The end goal must be nothing less than achieving a world where the suffering of all people—those with serious illness and those with opioid addiction—is alleviated through concerted, collaborative, multisector efforts that strive to realise a balanced approach to opioid medicines.

## *Panel:* Recommendations for WHO member states, international organisations, and local and global actors to realise opioid access without excess

- Advance collective responsibility of the global health system to reset norms such that safe access to pain relief is appropriately regarded as a public health priority and a key global public good championed by countries, international agencies such as WHO and the International Narcotics Control Board, and civil society organisations alike
- Expand the evidence-base for informative regulatory and clinical guidance on tackling the parallel crises of the pain pandemic and the opioid addiction epidemic by leveraging the expertise of the scientific and civil society communities, and addressing epistemic inequalities in the evidence base (eg, between high-income countries and low-income and middle-income countries, and disproportionate focus on opioid misuse and abuse)
- Provide technical support to WHO member states for modelling a balanced approach so that national level requests and recommendations related to the need for opioid medicines effectively seek to address the burden of serious health-related suffering within their countries while ensuring policies and resources to screen for and mitigate opioid misuse and diversion
- Secure local or regional manufacturing of off-patent, low-cost morphine based on WHO guidance^10^ to respond to existing supply gaps and related inequities accompanied by required guidelines for safe distribution and management and ongoing communication about processes to various regional offices and relevant ministries in member states
- Ensure informed medical decision making by the health workforce with the implementation of mandatory undergraduate training of skills and competencies necessary to prevent, assess, and treat pain and opioid use disorders
- Partner with local communities and well-established palliative care organisations and public health experts in opioid and substance addiction to strengthen advocacy at national, regional, and global levels—ensuring a society-wide endeavour to realise access without excess
- Merge efforts across actors and entities to highlight and disseminate freely available care models to reduce duplicative effort, promote timely uptake of available evidence, and encourage governments and health and education ministries to use these materials for licensing of health-care professionals and accreditation of schools and hospitals
- Integrate the need for a balanced approach throughout all evidence-based advocacy efforts, incorporating the knowledge and resources of well-established palliative care and substance abuse organisations with extensive, established networks and care delivery expertise at local and global levels
- Leverage and deepen partnerships between palliative care and prevention, addiction, and rehabilitative interest to ensure policies cohesively integrate balanced views into regulations and practices
- Build on civil society initiatives to dispel opioid myths and stigma among health professionals, policy makers, and the public
